# The mediating role of organizational justice in the relationship between cost consciousness and resource efficiency among human service professionals

**DOI:** 10.3389/fpsyg.2026.1903704

**Published:** 2026-10-02

**Authors:** Ammar Sevgili, Emin Yürekli, Mehmet Çanakci

**Affiliations:** 1 Department of Social Work, Faculty of Health Sciences, Sirnak University, Sirnak, Türkiye; 2 Department of Accounting and Taxation, Honaz Vocational School, Pamukkale Universitesi, Pamukkale, Türkiye; 3 Department of Public Finance, Faculty of Political Sciences, Ankara Yildirim Beyazit University, Ankara, Türkiye

**Keywords:** cost consciousness, mediation analysis, organizational justice perception, resource efficiency, social service management

## Abstract

**Background:**

Organizational psychology research suggests that employees’ perceptions of workplace practices play an important role in shaping organizational outcomes. In resource-constrained Human Service Organizations (HSOs), organizational justice may represent a key psychological mechanism linking cost-conscious practices to resource-efficient behaviors.

**Objective:**

This study examined whether organizational justice explains the association between cost consciousness and resource efficiency among professionals working in Human Service Organizations (HSOs).

**Methods:**

A cross-sectional survey design was employed with 246 professionals working in public and municipal human service institutions in Türkiye, including social workers, nurses, and administrative personnel. Data were analyzed using confirmatory factor analysis and regression-based mediation analysis with Hayes’ PROCESS Macro (Model 4) and 5,000 bootstrap resamples.

**Results:**

Cost consciousness was positively associated with both organizational justice perception (*β* = 0.500, *p* < 0.001) and resource efficiency (*β* = 0.466, *p* < 0.001). Organizational justice was also positively associated with resource efficiency (*β* = 0.207, *p* = 0.001). Mediation analysis indicated that organizational justice partially explained the association between cost consciousness and resource efficiency (indirect effect = 0.104, 95% CI [0.047–0.160]). The final model explained 37.7% of the variance in resource efficiency.

**Conclusion:**

The findings suggest that cost-conscious practices in human service settings may contribute more effectively to sustainable resource use when employees perceive organizational processes as fair and transparent. Strengthening organizational justice may therefore support workforce-related efficiency and sustainable service delivery in public and social care systems.

## 1 Introduction

Employees working in Human Service Organizations (HSOs) routinely operate in environments characterized by limited resources, increasing service demands, and heightened accountability expectations. In such settings, employees are expected not only to deliver continuous services to vulnerable populations but social workers, whose professional mandate explicitly includes advocacy for social justice within the institutions they serve (Gürhan Duran and Yurtsever, 2024) also to adapt to organizational practices aimed at maintaining efficiency and sustainability. Organizational psychology research suggests that employees’ perceptions of workplace practices play a critical role in shaping organizational attitudes, behavioral responses, and performance-related outcomes. Consequently, understanding how employees interpret resource-related managerial practices has become increasingly important for both organizational effectiveness and sustainable service delivery.

One of the organizational practices that has received growing attention is cost consciousness. Although often examined from a managerial and financial perspective, cost consciousness may also be understood as a cognitive and behavioral orientation reflecting employees’ awareness of resource limitations and their willingness to use organizational resources responsibly. In workforce-intensive service environments, employees’ interpretations of cost-related practices may influence not only organizational efficiency but also their attitudes toward organizational goals and resource stewardship. Accordingly, cost consciousness represents more than a financial control mechanism; it reflects an employee-centered organizational orientation that may shape how institutional resources are allocated, protected, and utilized in service systems.

The broader literature highlights an ongoing tension between efficiency and equity, particularly within social and public service organizations operating under marketization, outsourcing, and managerial reform processes. While cost-focused reforms may improve financial sustainability and institutional standardization (Bach-Mortensen and Barlow, 2021), they may also increase organizational pressure, workload, and uncertainty among employees, thereby affecting workforce capacity, service accessibility, and quality of care (Goodair et al., 2024). Furthermore, financial and institutional arrangements have been shown to influence critical social outcomes, including inequalities in service access and placement stability within child protection systems (Bach-Mortensen et al., 2023; Bach-Mortensen et al., 2025). These findings suggest that employees’ psychological responses to resource-related managerial practices may be as important as the practices themselves in shaping organizational outcomes.

Despite increasing interest in organizational efficiency and resource management, existing research has primarily focused on macro-level funding structures, administrative performance indicators, and institutional outcomes. Comparatively less attention has been paid to the psychological mechanisms through which employees interpret and respond to organizational efforts aimed at improving efficiency. In particular, limited empirical evidence exists regarding how employees evaluate resource allocation decisions as fair, transparent, and legitimate within organizational settings (Chen and Xu, 2020; Shonhadji, 2022). This gap is especially important because employee perceptions and organizational climate are widely recognized as fundamental determinants of workplace behavior, organizational commitment, and sustainable organizational functioning.

Beyond this practical gap, a more specific theoretical gap can be identified in the literature. Although Organizational Justice Theory and Social Exchange Theory jointly imply that fairness perceptions should translate cost-conscious managerial practices into resource-supportive employee behavior, this translational mechanism has rarely been tested as an explicit mediating pathway in resource-constrained public service settings; prior work has instead examined organizational justice predominantly as an antecedent of attitudinal outcomes such as trust, commitment, or turnover intention, rather than as a mechanism linking a managerial practice to a resource-related behavioral outcome (Stankevičiūtė and Savanevičienė, 2021; Moon et al., 2024). The present study addresses this unresolved theoretical issue by explicitly testing organizational justice perception as a mediating mechanism between cost consciousness and resource efficiency, thereby extending Organizational Justice Theory and Social Exchange Theory beyond their conventional attitudinal outcomes to a resource-behavioral outcome relevant to Human Service Organizations.

Within organizational psychology, organizational justice has emerged as one of the most influential constructs for understanding employees’ evaluations of workplace practices. Organizational justice refers to employees’ perceptions regarding the fairness of organizational procedures, resource distributions, and managerial interactions. Previous research consistently demonstrates that organizational justice is positively associated with organizational trust, work engagement, job satisfaction, organizational commitment, and various performance-related outcomes (Stankevičiūtė and Savanevičienė, 2021; Moon et al., 2024). Similarly, studies conducted in healthcare and public-sector contexts indicate that organizational justice contributes to employee engagement, readiness for organizational change, and positive workforce outcomes (Kebede and Wang, 2022; Zhou et al., 2022).

The relationship between organizational justice and employee behavior can be further understood through the lens of Social Exchange Theory. This perspective suggests that employees who perceive organizational processes as fair and respectful are more likely to reciprocate through positive workplace attitudes and behaviors that support organizational objectives. In this regard, fair and transparent organizational environments may strengthen employees’ willingness to internalize managerial practices, identify with organizational goals, and engage in responsible resource-use behaviors. Consistent with this argument, previous studies have suggested that organizational and motivational resources may mediate the effectiveness of managerial practices and organizational performance processes (Tantardini, 2022).

Drawing upon organizational justice literature and the broader organizational behavior perspective, the present study examines whether Organizational Justice Perception (OJP) explains the association between Cost Consciousness (CC) and Resource Efficiency (RE) among professionals working in Human Service Organizations. Specifically, the study adopts a managerial practice–employee perceptions–organizational outcomes framework, proposing that employees’ perceptions of fairness may represent an important psychological mechanism linking cost-conscious practices to resource-efficient behaviors. It is expected that cost consciousness will be associated with greater resource efficiency and that this relationship will be strengthened through employees’ perceptions of organizational justice.

The study contributes to the organizational psychology literature by demonstrating that resource efficiency in Human Service Organizations cannot be explained solely through technical or financial management mechanisms. Instead, the findings highlight the importance of employee perceptions, organizational justice, and workplace fairness as psychosocial factors that may support sustainable resource utilization and organizational effectiveness. By integrating organizational justice into the relationship between cost consciousness and resource efficiency, the study offers a psychologically grounded explanation of how managerial practices are translated into employee-supported organizational outcomes.

## 2 Theoretical and conceptual framework

### 2.1 Cost consciousness and resource efficiency in human service organizations

Human Service Organizations (HSOs) operate in environments where increasing service demands, workforce pressures, and limited institutional resources coexist. As organizations providing continuous services to disadvantaged and vulnerable populations, HSOs are required to balance institutional sustainability, service accessibility, and accountability under conditions of financial and operational constraint. In such settings, managerial decisions regarding the allocation and use of organizational resources influence not only financial efficiency but also employees’ workplace experiences, behavioral responses, and the continuity of service delivery.

Cost Consciousness (CC) refers to employees’ awareness of resource limitations and their tendency to consider the cost implications of organizational decisions and practices. In public and social care systems, cost consciousness should not be understood solely as expenditure reduction or budget control; rather, it reflects an organizational orientation that shapes how employees interpret and respond to resource-related managerial practices. Previous studies suggest that cost-focused managerial practices may strengthen accountability and (Lapuz et al., 2021; Tsyhaniuk and Akenten, 2025) contribute to more efficient resource allocation processes within organizations (Chen and Xu, 2020; Shonhadji, 2022). However, the literature also emphasizes that efficiency-oriented reforms implemented under marketization and outsourcing pressures may weaken organizational climate, increase workload, and negatively affect service quality in social and care systems (Bach-Mortensen and Barlow, 2021; Goodair et al., 2024).

Resource efficiency (RE), in the context of Human Service Organizations, refers to the effective and sustainable use of organizational resources in ways that support institutional objectives and service outcomes while minimizing unnecessary waste. In public service systems, efficiency cannot be reduced merely to producing more outputs with fewer resources. Rather, it also involves maintaining service quality, accessibility, and fairness in the distribution of limited resources according to social needs (Ruijer et al., 2022; Osei-Kojo, 2016). This perspective suggests that resource efficiency in HSOs is shaped not only by technical management practices but also by employees’ perceptions of organizational processes and workplace climate.

International research further demonstrates that financial and institutional regulations in care systems may directly influence inequalities in service access and critical social outcomes. Studies conducted in child protection and social care systems indicate that cost-oriented reforms may affect placement stability, workforce capacity, and the sustainability of service provision (Bach-Mortensen et al., 2023; Bach-Mortensen et al., 2025). These findings highlight that the relationship between cost consciousness and resource efficiency should be evaluated not only through managerial and financial mechanisms but also through the psychological and organizational conditions under which employees interpret and implement resource-related practices.

### 2.2 Organizational justice as a psychosocial organizational mechanism

Organizational justice refers to employees’ perceptions regarding the fairness, transparency, and legitimacy of organizational decisions, managerial practices, and resource distribution processes. In Human Service Organizations (HSOs), where institutional resources are limited and managerial decisions frequently involve prioritization and allocation processes, employees’ perceptions of fairness may play a critical role in shaping organizational attitudes and workplace behaviors. In workforce-intensive public and social care systems, organizational justice is considered an important psychosocial organizational mechanism influencing employee trust (Adam and Donelson, 2020), organizational commitment, work engagement, and performance-related outcomes.

The theoretical foundation of organizational justice is primarily grounded in Organizational Justice Theory and Social Exchange Theory. The organizational justice perspective suggests that employees who perceive organizational procedures and managerial decisions as fair are more likely to develop positive attitudes toward their organization and support institutional goals (Stankevičiūtė and Savanevičienė, 2021; Moon et al., 2024). Similarly, Social Exchange Theory argues that fair treatment strengthens reciprocal relationships between employees and organizations, encouraging employees to respond with behaviors that protect organizational resources and contribute to institutional effectiveness (Tantardini, 2022). Within this framework, organizational justice may strengthen employees’ willingness to internalize managerial practices and engage in more responsible and cooperative workplace behaviors.

Previous studies consistently demonstrate that organizational justice is associated with organizational trust, job satisfaction, organizational identification, employee engagement, turnover intention (Sökmen, 2020; Kahrıman et al., 2021), and performance-related attitudes in public institutions and care-oriented organizations. Research conducted with public employees indicates that fair reward systems, transparent managerial processes, and respectful communication contribute to organizational sustainability and positive workforce outcomes (Moon et al., 2024). Similarly, studies focusing on performance management practices show that transparent goal setting, monitoring, and feedback processes may strengthen employees’ perceptions of fairness and increase work commitment (Stankevičiūtė and Savanevičienė, 2021; Kang et al., 2021). Longitudinal evidence further suggests that organizational justice may influence employee attitudes through intermediate psychosocial processes such as organizational identification (Ho, 2024).

Recent studies in healthcare and public-sector settings similarly demonstrate that organizational justice is closely associated with employee engagement, readiness for organizational change, perceived organizational support, and turnover-related outcomes (Kebede and Wang, 2022; Zhou et al., 2022). Moreover, fairness perceptions have been linked to organizational learning, feedback culture, employee voice behaviors, and reduced organizational silence in public institutions (Burton and Boyd, 2024; Marzec, 2023; Jeon and Jeong, 2024; Mok and Leong, 2021). These findings suggest that employees are more likely to accept and support managerial practices when organizational decisions are perceived as fair, transparent, and legitimate.

This mediating function of organizational justice is further supported by evidence from other institutional contexts. Organizational justice has been shown to interact with organizational learning culture and job embeddedness in shaping employees’ turnover-related intentions (Saoula et al., 2019a), while organizational citizenship behavior has been identified as a behavioral pathway through which organizational justice affects turnover intention (Saoula et al., 2019b). These findings reinforce the view that organizational justice is not merely an outcome of managerial practices but can also function as an explanatory mechanism that channels such practices into concrete behavioral consequences.

In Human Service Organizations, where employees directly interact with vulnerable populations and institutional resources are closely tied to service continuity, organizational justice may represent a critical psychological mechanism linking managerial practices to resource-related organizational outcomes. Specifically, when cost-conscious managerial decisions are perceived as fair and justified, employees may be more willing to protect organizational resources, avoid waste, and support efficient resource utilization processes. Therefore, organizational justice may help explain how cost consciousness is translated into resource efficiency through employees’ perceptions and workplace behaviors within public and social care systems.

Recent empirical investigations further substantiate the behavioral consequences of organizational justice in public and service-oriented sectors. For instance, Jabeen et al. (2021) demonstrated that fairness in organizational appraisals significantly enhances employees’ job performance and organizational citizenship behaviors (OCB) through increased work engagement. Similarly, empirical evidence from the public sector indicates that justice perceptions directly foster interpersonal trust, which in turn elevates overall employee satisfaction and cooperative workplace dynamics (Alotaibi, 2024). Furthermore, organizational fairness has been identified as a critical component of human resource practices that drive sustainable job performance in public administration (Ouabi et al., 2024). Collectively, these empirical findings illustrate that when employees perceive their work environment as just, they do not merely experience higher satisfaction; they actively adjust their work behaviors to support organizational efficiency and collective goals.

### 2.3 Conceptual model and hypotheses

The present study is grounded in organizational justice and organizational behavior literature, which emphasizes that employees’ perceptions of workplace practices play a critical role in shaping organizational attitudes and behavioral outcomes. According to Organizational Justice Theory, employees evaluate managerial decisions not only in terms of their outcomes but also according to the perceived fairness and legitimacy of organizational procedures. Similarly, Social Exchange Theory suggests that employees who perceive fair treatment from their organization are more likely to reciprocate through positive workplace attitudes and behaviors that support organizational goals (Tantardini, 2022). To date, empirical studies have primarily examined these constructs either in isolation or through pairwise relationships. For example, previous research has investigated the relationship between managerial cost practices and efficiency outcomes (Chen and Xu, 2020; Bhutoria and Aljabri, 2022), as well as the impact of organizational justice on employee performance and engagement (Moon et al., 2024; Kebede and Wang, 2022). However, empirical research examining the simultaneous relationship among cost consciousness, organizational justice, and resource efficiency remains limited.

Within Human Service Organizations (HSOs), employees operate under conditions characterized by resource constraints, increasing service demands, and accountability pressures. In such environments, cost-conscious managerial practices may influence employees’ perceptions of how organizational resources are managed and distributed. Previous studies suggest that managerial practices emphasizing accountability and resource awareness may contribute to organizational effectiveness when employees perceive these practices as legitimate and fair (Chen and Xu, 2020; Whitebread et al., 2022; Bhutoria and Aljabri, 2022). Consequently, employees’ perceptions of organizational justice may represent an important psychological mechanism through which cost-conscious practices influence workplace outcomes.

The organizational justice literature further indicates that fair and transparent organizational environments strengthen employee trust, organizational identification, engagement, and commitment to organizational objectives (Stankevičiūtė and Savanevičienė, 2021; Moon et al., 2024). Employees who perceive organizational decisions as fair may therefore be more willing to support organizational goals, protect institutional resources, and engage in behaviors that contribute to efficient resource utilization. Consistent with this perspective, previous research suggests that organizational and motivational resources may mediate the relationship between managerial practices and organizational outcomes (Tantardini, 2022).

The decision to conceptualize organizational justice specifically as the mediating mechanism, rather than treating it merely as a correlate of cost-conscious practices, is further supported by studies that have modeled organizational justice as an explicit mediator of behavioral outcomes. For instance, Saoula et al. (2019b) demonstrated that organizational justice affects turnover intention indirectly through organizational citizenship behavior, indicating that fairness perceptions translate into concrete workplace behaviors rather than remaining purely attitudinal. Applying this logic to the present context, organizational justice is expected to function as the psychological channel through which cost-conscious managerial practices are converted into resource-efficient employee behavior, rather than merely co-occurring with it.

Based on this theoretical framework, the present study proposes that employees with higher levels of cost consciousness will report higher levels of resource efficiency. Furthermore, organizational justice is expected to explain this relationship by increasing employees’ acceptance and internalization of cost-related managerial practices. Accordingly, organizational justice is conceptualized as a psychosocial mechanism linking cost consciousness to resource efficiency among professionals working in Human Service Organizations.

Based on the theoretical and empirical literature, the following hypotheses were developed:

*H1*: Cost consciousness is positively associated with resource efficiency among professionals working in Human Service Organizations.

*H2*: Cost consciousness is positively associated with organizational justice perception among professionals working in Human Service Organizations.

*H3*: Organizational justice perception is positively associated with resource efficiency among professionals working in Human Service Organizations.

*H4*: Organizational justice explains the association between cost consciousness and resource efficiency among professionals working in Human Service Organizations.

The conceptual model is shown in Figure 1.

**Figure 1:**
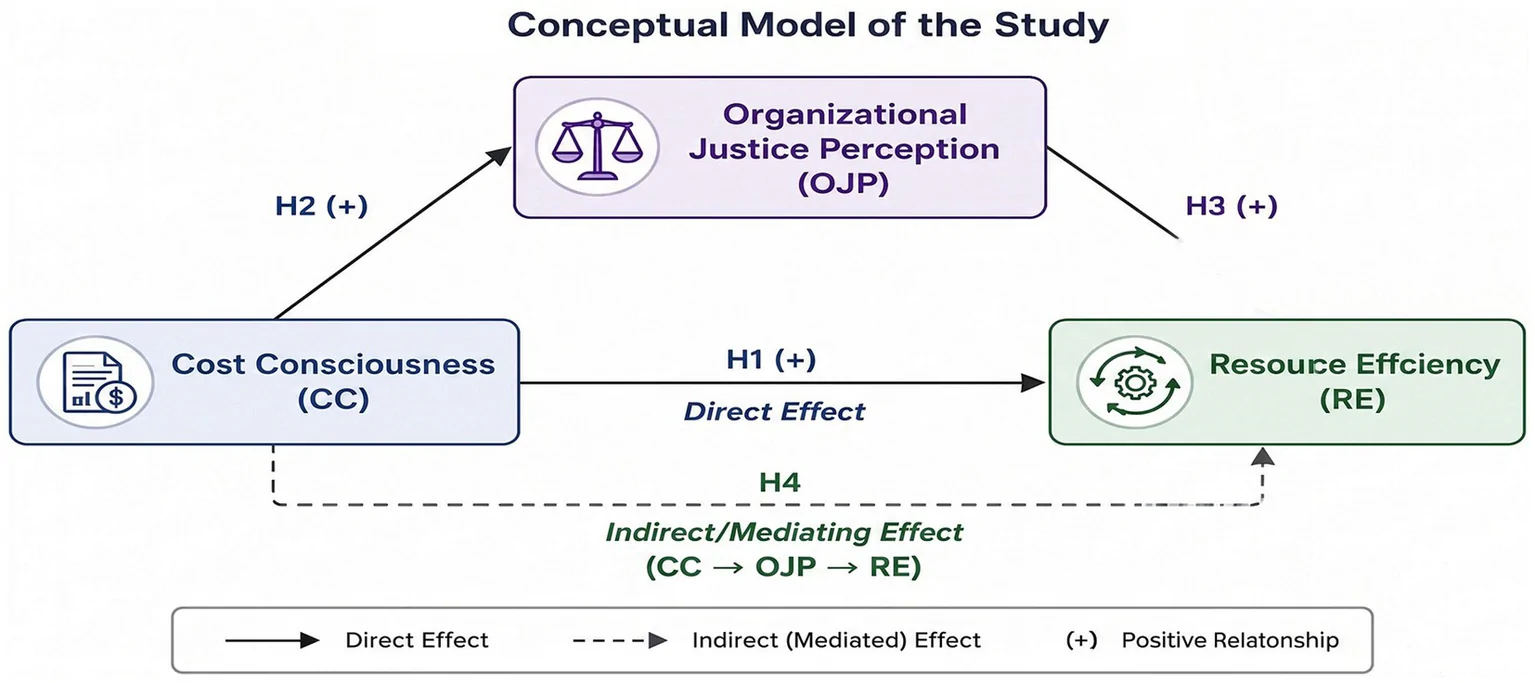
Conceptual model of the study.

## 3 Materials and methods

### 3.1 Study design

This study employed a quantitative, cross-sectional survey design based on a correlational research model. The study examined the associations between cost consciousness, resource efficiency, and organizational justice perception among personnel working in Human Service Organizations (HSOs). In addition, the explanatory role of organizational justice perception in the association between cost consciousness and resource efficiency was tested through regression-based mediation analysis. No experimental intervention was conducted, and all variables were measured within a single data collection period.

### 3.2 Participants and setting

The study sample consisted of 246 employees working in Human Service Organizations. Of the participants, 56.9% were male and 43.1% were female. Regarding age, 32.1% were aged 20–29, 29.7% were aged 30–39, 15.0% were aged 40–49, and 23.2% were aged 50 years and above. In terms of education, the majority were university graduates (56.5%), followed by high school graduates (24.8%), associate degree graduates (10.6%), and master’s degree graduates (8.1%).

Regarding professional positions, social workers constituted the largest group (39.0%), followed by nurses (22.8%) and managerial/administrative personnel (19.9%). Participants were mainly employed in institutions affiliated with the Ministry of Family and Social Services (32.9%), municipal social service units (31.7%), and nursing homes, care, and rehabilitation centers (23.6%). More than half of the participants (56.9%) had 10 years or more of professional experience. In addition, 64.2% reported having no knowledge of institutional financial matters, while 58.5% stated that they had not received any training on cost management or resource efficiency.

### 3.3 Measures

Data were collected using the Cost Consciousness Scale (CC), the Resource Efficiency Scale (RE), and the Organizational Justice Perception Scale (OJP). The measurement instruments were developed based on an extensive review of the relevant literature and the conceptual definitions of each construct. The item development process aimed to capture employees’ perceptions of cost consciousness, resource efficiency, and organizational justice within Human Service Organizations.

Initially, an item pool was generated for each construct following a review of previous theoretical and empirical studies. To evaluate content validity, the preliminary items were reviewed by five academics with expertise in social work, public administration, health management, and measurement and evaluation. Based on expert feedback, revisions were made to improve conceptual clarity, content relevance, and linguistic consistency. Items that were considered redundant or insufficiently representative of the target construct were revised before finalization.

“Because existing scales in the literature primarily focus on corporate financial accounting rather than psychosocial cost awareness in social services, all three instruments used in this study (the Cost Consciousness Scale, the Resource Efficiency Scale, and the Organizational Justice Perception Scale) were developed originally by the authors as *de novo* measurement tools specifically tailored for the HSO context.

The scale development process followed a systematic approach. Initially, an extensive literature review was conducted to generate a preliminary item pool that captured the unique psychosocial dynamics of resource management in public service settings. To establish content and face validity, the preliminary item pool was submitted to an expert panel consisting of five academics with domain expertise in social work, public administration, health management, and psychometrics. The experts evaluated each item for conceptual clarity, construct relevance, and linguistic appropriateness. Based on their qualitative feedback, ambiguous items were revised, and redundant items were eliminated to form the final measurement instruments. The complete set of items for each scale is provided in Appendix A to ensure methodological transparency and replicability.”

To examine the psychometric properties of the measurement instruments, exploratory factor analysis (EFA) and confirmatory factor analysis (CFA) were conducted. Prior to factor analysis, Kaiser–Meyer–Olkin (KMO) coefficients and Bartlett’s tests of sphericity were examined to assess sampling adequacy and the suitability of the data for factor analysis. The findings indicated that the data were appropriate for factor analytic procedures. Internal consistency reliability was evaluated using Cronbach’s alpha coefficients.

The factor structures of the scales, item factor loadings, communalities, item–total correlations, and reliability coefficients are presented in Table 1. The findings demonstrated that all scales exhibited satisfactory psychometric properties, supporting their use for assessing the study variables among professionals working in Human Service Organizations.

**Table 1:** Exploratory factor analysis and reliability results of the scales used in the study (*n* = 246).

Item	Factor loadings	Common variance (h^2^)	Item–total correlation
A. Cost consciousness scale			
CC1	0.772	0.597	0.722
CC2	0.825	0.680	0.784
CC3	0.871	0.758	0.834
CC4	0.895	0.801	0.864
CC5	0.897	0.805	0.864
CC6	0.876	0.768	0.838
CC7	0.867	0.752	0.831
CC8	0.803	0.645	0.753
CC9	0.747	0.558	0.685
CC10	0.775	0.600	0.724
B. Resource efficiency scale			
RE1	0.838	0.703	0.794
RE2	0.828	0.686	0.782
RE3	0.847	0.717	0.803
RE4	0.820	0.672	0.774
RE5	0.829	0.687	0.786
RE6	0.836	0.698	0.794
RE7	0.845	0.714	0.805
RE8	0.862	0.744	0.828
RE9	0.870	0.757	0.836
RE10	0.750	0.563	0.694
C. Organizational justice perception scale			
OJP1	0.841	0.708	0.796
OJP2	0.869	0.755	0.828
OJP3	0.855	0.731	0.812
OJP4	0.872	0.761	0.836
OJP5	0.890	0.793	0.859
OJP6	0.886	0.785	0.855
OJP7	0.902	0.813	0.874
OJP8	0.886	0.785	0.857
OJP9	0.851	0.725	0.814

KMO = 0.912; Bartlett’s Sphericity Test: χ^2^(45) = 2367.168; *p* < 0.001; Total explained variance = 69.64%; Cronbach’s Alpha = 0.950.

KMO = 0.907; Bartlett’s Sphericity Test: χ^2^(45) = 2244.779; *p* < 0.001; Total explained variance = 69.42%; Cronbach’s Alpha = 0.951.

KMO = 0.916; Bartlett’s Sphericity Test: χ^2^(36) = 2465.749; *p* < 0.001; Total explained variance = 76.17%; Cronbach’s Alpha = 0.961.

The findings presented in Table 1 show that all three scales exhibit a single-factor structure and possess strong psychometric properties. The fact that the total variance ratios explained in the Cost Consciousness Scale and the Resource Efficiency Scale are approximately 69%, and over 76% in the Organizational Justice Perception Scale, reveals that the scales explain the structures they aim to measure at a high level. It was determined that the factor loadings in all scales were above acceptable limits, the common variance (h^2^) values were at sufficient levels, and the item–total correlations were high. Furthermore, Cronbach’s alpha coefficients being above 0.95 indicate that the scales have very high internal consistency. Since removing any item from the scale did not significantly increase the reliability coefficients, it was decided to retain all items in the scale. Based on these findings, the scales used in the study were accepted as valid and reliable measurement tools.

Furthermore, confirmatory factor analysis was applied to validate the measurement structures of the Cost Consciousness Scale, Resource Efficiency Scale, and Organizational Justice Perception Scale used in the study. Single-factor models proposed based on the theoretical framework were tested for each scale; construct validity was assessed through model fit statistics and standardized regression weights. Diagrams related to confirmatory factor analysis are presented in Figure 2, and summary findings of the goodness-of-fit indices are presented in the table.

**Figure 2:**
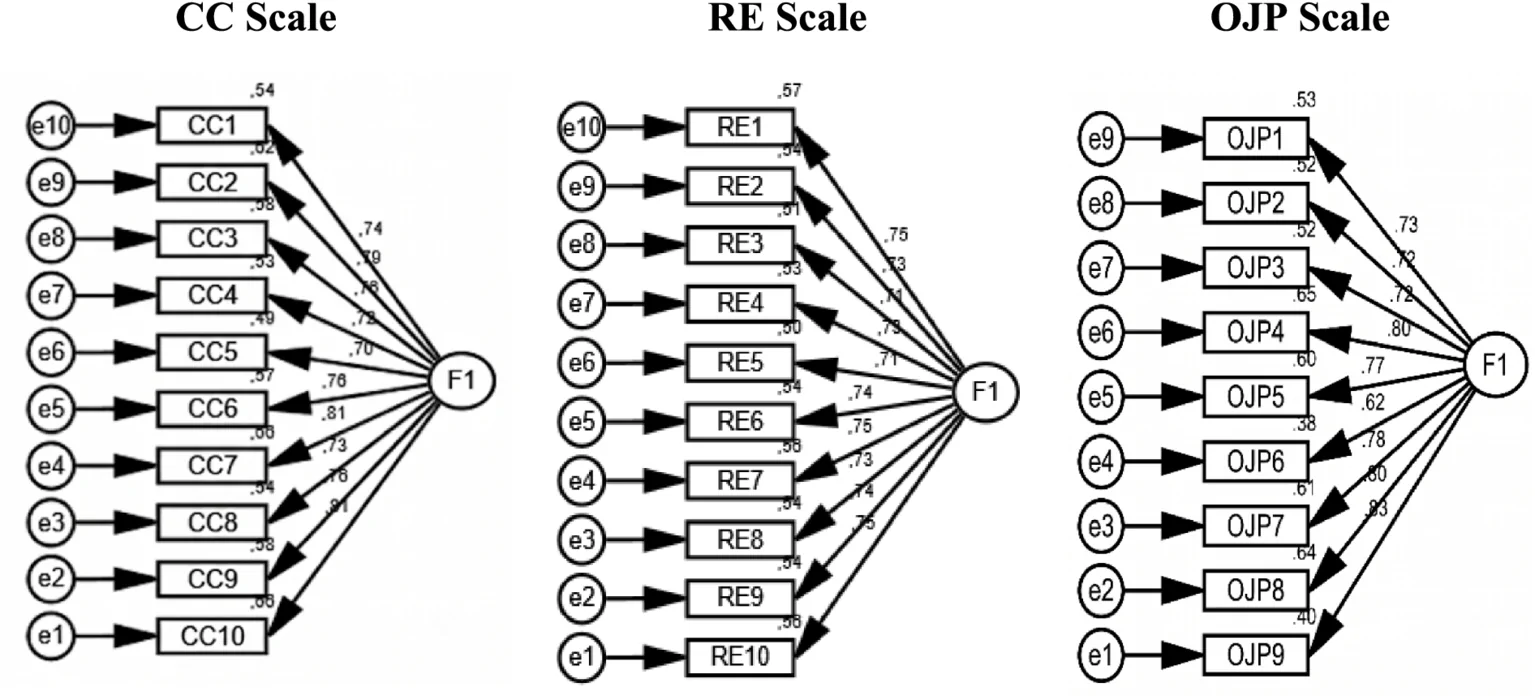
Confirmatory factor analysis diagrams.

When examining the confirmatory factor analysis results, it is seen that the goodness-of-fit indices obtained for all three scales indicate that the measurement models fit the data well. The fact that the ratios of chi-square values to degrees of freedom are within acceptable limits (χ^2^/df = 1.336–1.840) indicates that the overall fit of the models is adequate and good (Kline, 2016). The fact that the RMR values for residual errors are within acceptable ranges for all models suggests that measurement errors are low.

When comparative and absolute fit indices are evaluated together, it is observed that the GFI, NFI, and RFI values are above the recommended thresholds in all three scales, while the IFI, TLI, and CFI values indicate a high level of fit (Hu and Bentler, 1999; Hooper et al., 2008). The RMSEA values ranging from 0.037 to 0.059 support that the approximate error levels are low and within acceptable limits; thus, the measurement models will also show good fit at the population level (Table 2).

**Table 2:** Fit indices.

Fit indices	cc scale	RE scale	OJP scale	Threshold values	Source
χ^2^	46.758	64.417	47.454		
df	35	35	27		
χ^2^/df (CMIN/DF)	1.336	1.840	1.758	0 ≤ χ^2^/df ≤ 5	Kline (2016)
RMR	0.049	0.060	0.060	0.05 ≤ RMR ≤ 0.08	Hooper et al. (2008)
GFI	0.964	0.950	0.959	0.80 ≤ GFI ≤ 0.95	Kline (2016)
NFI	0.969	0.952	0.960	0.90 ≤ NFI ≤ 1.00	Hu and Bentler (1999)
RFI	0.960	0.938	0.947	0.90 ≤ RFI ≤ 1.00	Hu and Bentler (1999)
IFI	0.992	0.977	0.982	0.80 ≤ IFI ≤ 1.00	Hooper et al. (2008)
TLI	0.990	0.971	0.976	0.80 ≤ TLI ≤ 1.00	Hooper et al. (2008)
CFI	0.992	0.977	0.982	0.90 ≤ CFI ≤ 1.00	Hu and Bentler (1999)
RMSEA	0.037	0.059	0.056	0.05 ≤ RMSEA ≤ 0.08	Hu and Bentler (1999)

When examining the standardized regression weights, the items of the Cost Consciousness Scale represented the factor at a medium-high and high level (0.698–0.813), the factor loadings of the Resource Efficiency Scale items were consistent and adequate (0.710–0.754), and the items of the Organizational Justice Perception Scale showed moderate to high correlations with the factor (0.620–0.805). No item was found to have a low factor loading or a structure that weakened the fit of the measurement model.

When these findings are evaluated together, it can be concluded that the single-factor structures of the three scales used in the study are confirmed, the measurement models are consistent with the data, and the scales represent the relevant theoretical structures in a valid and reliable manner.

### 3.4 Statistical analysis

The data obtained from the research were analyzed using the IBM SPSS Statistics 22.0 software package in a computer environment. Before proceeding to the analysis process, checks for missing data and outliers were performed on the variables in the dataset, and missing data were excluded from the analysis on a listwise basis. The normality of the variables was assessed by examining skewness and kurtosis values. The obtained values were found to be within acceptable limits, and it was decided to use parametric analyses.

Because all study variables were collected using self-report questionnaires administered at a single point in time, the potential risk of common method bias was assessed using Harman’s single-factor test. An unrotated exploratory factor analysis was conducted including all measurement items. The results indicated that the first unrotated factor accounted for less than 50% of the total variance, suggesting that common method bias was unlikely to pose a serious threat to the validity of the findings.

Within the scope of descriptive statistics, frequencies and percentages were calculated for the sociodemographic characteristics of the participants, while arithmetic means and standard deviation values were calculated for the research variables. To evaluate the psychometric properties of the Cost Consciousness Scale, Resource Efficiency Scale, and Organizational Justice Perception Scale used in the study, Exploratory Factor Analysis (EFA) was applied separately for each scale. Sample adequacy was tested using the Kaiser–Meyer–Olkin (KMO) coefficient and Bartlett’s sphericity test. The internal consistency of the scales was evaluated by calculating Cronbach’s alpha coefficient.

Pearson correlation analysis was performed to determine the relationships between the study’s key variables. Regression-based mediation analysis was conducted to examine the mediating role of Organizational Justice Perception in the relationship between Cost Consciousness and Resource Efficiency. To test the mediation effect, which is the main hypothesis of the study, the regression-based Process Macro (Model 4) developed by Hayes (2018) was used. The statistical significance of indirect effects was evaluated based on the bootstrap method with 5,000 resampling values and 95% confidence interval (CI) values, in line with current approaches in the literature. A significance level of *p* < 0.05 was accepted in all statistical analyses.

## 4 Results

### 4.1 Descriptive statistics and correlations

Descriptive statistics and Pearson correlation coefficients for the main study variables are presented in Table 3.

**Table 3:** Descriptive statistics and Pearson correlation analysis results for cost consciousness, resource efficiency, and organizational justice perception variables.

Variable	Mean	Std. Dev.	Skewness	Kurtosis	CC	RE	OJP
CC	3.783	0.858	0.682	1.052	1.000		
RE	4.031	0.682	0.896	1.125	0.587**	1.000	
OJP	2.435	0.874	0.369	−0.536	0.500**	0.449**	1.000

* < 0.05; ** < 0.01; Pearson Correlation Analysis. OJP; Organizational Justice Perception, RE; Resource Efficiency, CC; Cost Consciousness.

Pearson correlation analysis was performed to determine the relationships between the key variables included in the study (Table 3). According to the analysis results, the average cost consciousness level of employees was determined to be 3.78 ± 0.86, the average resource efficiency perception was 4.03 ± 0.68, and the average organizational justice perception was 2.44 ± 0.87. The correlation analysis findings show that there is a moderate, positive, and statistically significant relationship between cost consciousness and resource efficiency (*r* = 0.587; *p* < 0.01). Similarly, a moderate, positive, and significant relationship was found between cost consciousness and Organizational Justice Perception (*r* = 0.500; *p* < 0.01). Furthermore, a positive and significant relationship was found between resource efficiency and Organizational Justice Perception (*r* = 0.449; *p* < 0.01).

A regression-based mediation analysis was conducted to examine the mediating role of Organizational Justice Perception (OJP) in the relationship between Cost Consciousness (CC) and Resource Efficiency (RE) (Table 4). According to the analysis results, CC was found to have a positive and statistically significant effect on OJP (*β* = 0.500; *p* < 0.001), and this model explains 25% of the variance (R^2^ = 0.250). When examining the total effect of CC on RE, a positive and significant relationship was observed between the variables (*β* = 0.466; *p* < 0.001; R^2^ = 0.345). When the mediating variable OJP was included in the model, it was found that the direct effect of CC on RE decreased but remained significant (*β* = 0.384; *p* < 0.001) and that OJP had a positive and significant effect on RE (*β* = 0.207; *p* = 0.001). The total variance explained in this model increased to 37.7% (R^2^ = 0.377). The indirect effect coefficient calculated using the bootstrap method was found to be significant (*β* = 0.104; 95% CI [0.047–0.160]) and its confidence interval did not include the zero value. The findings indicate that Organizational Justice Perception plays a partial mediating role in the relationship between CC and RE (Figure 3).

**Table 4:** Regression and mediation analysis results regarding the mediating role of organizational justice perception (OJP) in the relationship between CC and RE.

Dependent variable	Independent variable	*ß*	SE	*t*	*p*	95% CI lower	95% CI upper
OJP	CC (a)	0.500	0.057	9.011	**0.000**	0.398	0.621
R = 0.500; R-sq = 0.250; *F* = 81.201; *p* = 0.000							
RE	CC (c)	0.587	0.041	11.323	**0.000**	0.385	0.548
R = 0.587; R-sq = 0.345; *F* = 128.218; *p* = 0.000							
RE	CC (c’)	0.483	0.047	8.268	**0.000**	0.293	0.476
	OJP (b)	0.207	0.046	3.541	**0.001**	0.072	0.251
R = 0.614; R-sq = 0.377; *F* = 73.411; *p* = 0.000							
Total effect		0.466	0.041	11.323	0.000	0.385	0.548
Direct effect		0.384	0.047	8.268	0.000	0.293	0.476
Indirect effect		0.104	0.028	-	-	0.047	0.160

Bold values indicate statistically significant path coefficients (*p* < .05).

**Figure 3:**
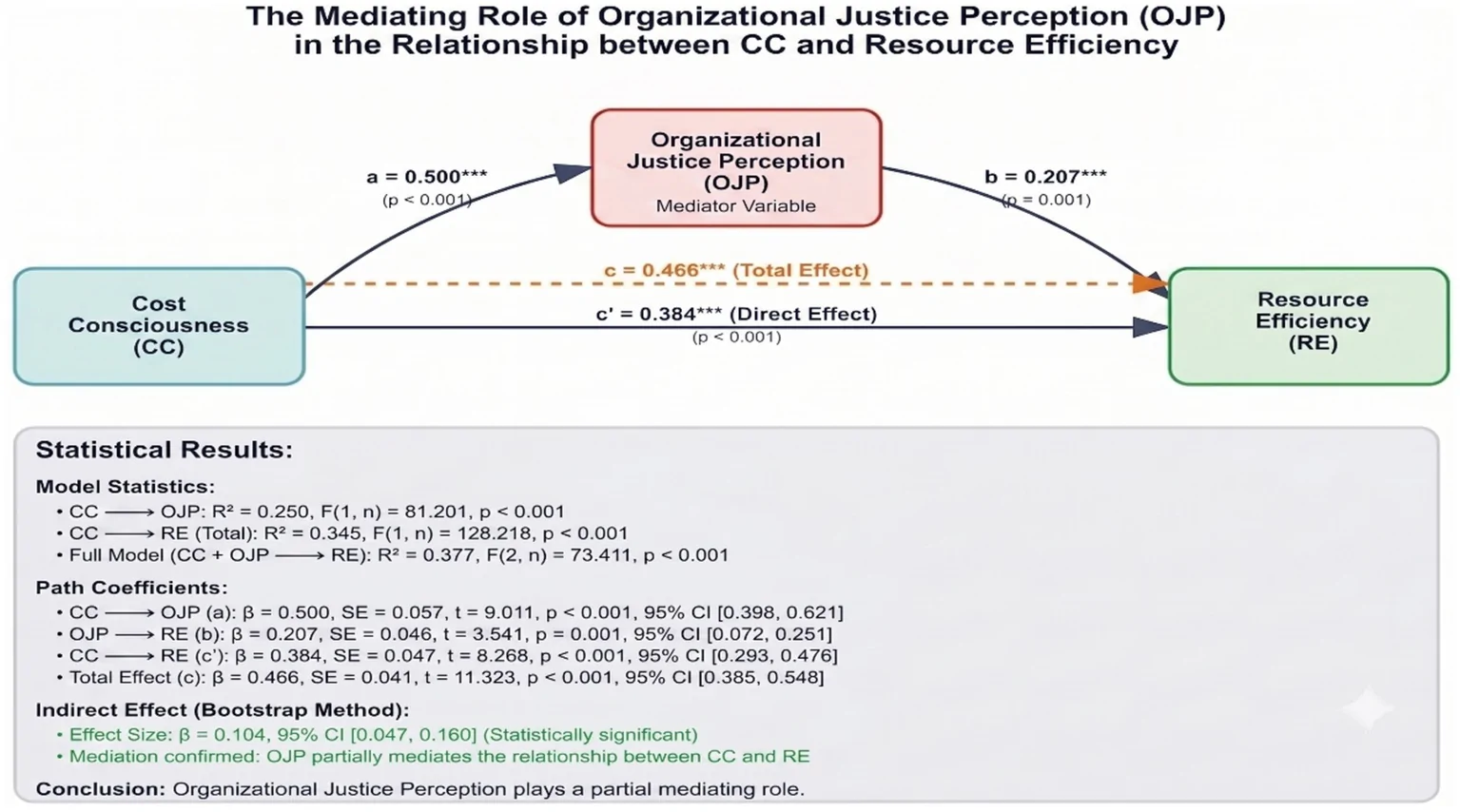
Regression and mediation analysis results regarding the mediating role of organizational justice perception (OJP) in the relationship between CC and RE.

## 5 Discussion

The present study examined the associations between cost consciousness (CC), resource efficiency (RE), and organizational justice perception (OJP) among professionals working in Human Service Organizations (HSOs). The findings demonstrated that cost consciousness was positively associated with both organizational justice perception and resource efficiency. In addition, organizational justice perception explained the association between cost consciousness and resource efficiency, supporting its role as a psychosocial organizational mechanism within Human Service Organizations.

The positive association between cost consciousness and resource efficiency suggests that awareness regarding institutional costs and resource limitations may support more careful, accountable, and purposeful use of organizational resources in public and social care settings. This finding is consistent with previous studies indicating that managerial practices emphasizing accountability and resource awareness are associated with organizational efficiency and performance outcomes in public institutions (Mostafa et al., 2015; Tantardini, 2022). However, the present findings also suggest that the relationship between cost consciousness and efficiency cannot be understood solely through technical or financial processes. Instead, organizational and psychosocial conditions appear to shape how employees interpret and implement resource-related managerial practices.

One of the central findings of the study is that organizational justice perception explained the association between cost consciousness and resource efficiency. Specifically, the findings suggest that cost-conscious managerial practices may contribute more effectively to resource efficiency when employees perceive organizational decisions and resource allocation processes as fair and transparent. This finding supports organizational justice literature emphasizing that employees’ fairness perceptions influence organizational trust, work engagement, and organizational commitment (Stankevičiūtė and Savanevičienė, 2021; Moon et al., 2024). In workforce-intensive Human Service Organizations, employees who perceive organizational practices as legitimate and transparent may be more willing to support institutional goals and protect organizational resources.

The findings further indicate that organizational justice perception was positively associated with resource efficiency. This result suggests that efficiency in Human Service Organizations may depend not only on structural and managerial arrangements but also on employees’ psychosocial evaluations of organizational climate. Previous studies similarly report that fair organizational environments strengthen employees’ organizational identification, responsibility perceptions, and positive workplace behaviors (Çakıroğlu, 2022; Atikbay and Öner, 2020; Çakıroğlu and Deli, 2023). Within public and social care systems, where institutional resources are directly linked to service continuity and vulnerable populations, organizational justice may therefore represent an important organizational condition supporting sustainable resource utilization.

The mediation analysis demonstrated that organizational justice perception partially explained the association between cost consciousness and resource efficiency. The persistence of the direct association after including organizational justice in the model suggests that additional organizational and institutional mechanisms may also contribute to resource efficiency, including professional responsibility, organizational procedures, and institutional accountability structures. Nevertheless, the statistically significant indirect effect highlights that employees’ fairness perceptions represent an important explanatory mechanism linking managerial practices to organizational outcomes in Human Service Organizations.

### 5.1 Theoretical implications

From a theoretical standpoint, the findings extend Organizational Justice Theory and Social Exchange Theory by demonstrating that organizational justice can operate as a mediating psychosocial mechanism that translates a managerial practice — cost consciousness — into a resource-related behavioral outcome, rather than functioning solely as an antecedent of attitudinal outcomes such as trust or commitment. This contributes to the organizational psychology literature by positioning fairness perceptions as an explanatory pathway linking managerial practices to sustainable resource-use behaviors in Human Service Organizations, an outcome domain that has received comparatively little attention in prior organizational justice research.

### 5.2 Practical implications

From a practical perspective, the findings suggest that cost-conscious managerial practices may be more sustainable and effective when implemented within transparent and participatory organizational environments. Rather than communicating cost-related decisions solely as financial necessities, managers in Human Service Organizations may strengthen employees’ acceptance of such practices by supporting procedural transparency (Zelnick et al., 2021) and organizational fairness. In this respect, the study contributes to public and social care management literature by suggesting that efficiency and fairness should not be viewed as competing organizational goals but as complementary organizational processes supporting sustainable service delivery.

### 5.3 Limitations and future research directions

Several limitations of this study should be acknowledged. First, the study employed a cross-sectional design, which limits causal interpretation of the associations between cost consciousness, organizational justice perception, and resource efficiency. Second, the data were collected using self-report measurement tools, which may increase the risk of common method bias and social desirability effects. Third, the sample consisted of professionals working in specific public and municipal Human Service Organizations in Türkiye, which may limit the generalizability of the findings to different institutional and cultural settings.

Future studies may benefit from longitudinal and mixed-method research designs to better examine the temporal and contextual dynamics of organizational justice and resource-related organizational behaviors. In addition, incorporating leadership styles, organizational culture, and employee wellbeing variables into future models (Almeida Marques, 2025) may contribute to a more comprehensive understanding of workforce sustainability and organizational efficiency in Human Service Organizations.

Although mediation analysis was employed to examine the proposed relationships, the cross-sectional nature of the data does not allow causal inferences regarding the observed indirect effects. Therefore, the findings should be interpreted as evidence of statistical mediation rather than causal mediation. Future longitudinal and experimental studies are needed to establish the temporal ordering of the proposed relationships.

## 6 Conclusion

This study demonstrated that cost consciousness was positively associated with resource efficiency among professionals working in Human Service Organizations and that organizational justice perception explained this association. The findings suggest that resource efficiency in public and social care systems cannot be understood solely through technical or financial management approaches. Instead, fair, transparent, and participatory organizational environments may represent important psychosocial conditions supporting sustainable resource utilization and service continuity.

Accordingly, cost-conscious managerial practices in Human Service Organizations may become more effective when employees perceive organizational processes as legitimate and fair. In workforce-intensive public service systems, strengthening organizational justice may therefore contribute not only to institutional efficiency but also to sustainable service delivery and organizational sustainability.

## Data Availability

The raw data supporting the conclusions of this article will be made available by the authors, without undue reservation.

## References

[ref1] Adam M. B. Donelson A. J. (2020). Trust is the engine of change: a conceptual model for trust building in health systems. Syst. Res. Behav. Sci. 39, 116–127. doi: 10.1002/sres.2766

[ref22] Almeida Marques P. C. (2025). Strategic resilience in healthcare: towards a digitalised and regenerative balanced scorecard framework. J. Health Organiz. Manage. 1–24. doi: 10.1108/JHOM-09-2025-0595 42252543

[ref2] Alotaibi M. (2024). The role of trust in supervisors as a mediator between fairness of appraisal evaluation and employee satisfaction: evidence from the public sector in Saudi Arabia. JoMW 2024, 375–385. doi: 10.53935/jomw.v2024i4.1134

[ref3] Atikbay T. Öner Y. (2020). Effects of human resources management practices and organizational justice perceptions on organizational cynicism: a research on municipalities in a developing country. Manag. Sci. Lett. 10, 1659–1670. doi: 10.5267/j.msl.2020.1.014

[ref4] Bach-Mortensen A. M. Barlow J. (2021). Outsourced austerity or improved services? A systematic review and thematic synthesis of the experiences of social care providers and commissioners in quasi-markets. Soc. Sci. Med. 276:113844. doi: 10.1016/j.socscimed.2021.113844, 33773477

[ref5] Bach-Mortensen A. M. Goodair B. Barlow J. (2023). For-profit outsourcing and its effects on placement stability and locality for children in care in England, 2011–2022: a longitudinal ecological analysis. Child Abuse Negl. 144:106245. doi: 10.1016/j.chiabu.2023.106245, 37258367 PMC10933776

[ref6] Bach-Mortensen A. Goodair B. Degli Esposti M. Needham C. (2025). England’s two tier care system deepens social care inequalities. BMJ 390:e084808. doi: 10.1136/bmj-2025-084808, 40664438

[ref7] Bhutoria A. Aljabri N. (2022). Managerial practices and school efficiency: a data envelopment analysis across OECD and MENA countries using TIMSS 2019 data. Large-Scale Assess. Educ. 10:24. doi: 10.1186/s40536-022-00147-3

[ref8] Burton J. Boyd M. (2024). Improving the climate of organizational work units through socially constructed performance appraisals. Pinnacle 2. doi: 10.61643/c35090

[ref9] Çakıroğlu D. (2022). Organizational silence’s mediation impact on the effect of organizational justice’s on intention to quit. J. Bus. Res.-Turk 14, 219–231. doi: 10.20491/isarder.2022.1376

[ref10] Çakıroğlu C. Deli M. (2023). The consequences of nepotistic attitudes and behaviors of educational institution managers. Int. J. Soc. Sci. 7, 170–184. doi: 10.52096/usbd.7.28.12

[ref11] Chen C. Xu C. (2020). No, I cannot just walk away’: government career entrenchment in China. Int. Rev. Adm. Sci. 87, 944–961. doi: 10.1177/0020852319884624

[ref12] Goodair B. McManus A. Degli Esposti M. Bach-Mortensen A. (2024). How outsourcing has contributed to England’s social care crisis. BMJ 387:e080380. doi: 10.1136/bmj-2024-080380, 39537332 PMC11577636

[ref13] Gürhan Duran E. Yurtsever E. (2024). Examination of social justice advocacy by social workers. J. Soc. Soc. Work 35, 269–290. doi: 10.33417/tsh.1324204

[ref14] Hayes A. F. (2018). Introduction to Mediation, Moderation, and Conditional process Analysis: A Regression-based Approach. New York, NY: The Guilford Press.

[ref15] Ho H. C. Y. (2024). A one-year prospective study of organizational justice and work attitudes: an extended job demands-resources model. J. Manager. Psychol. 40, 21–36. doi: 10.1108/JMP-02-2024-0113

[ref9003] Hooper D. Coughlan J. Mullen M. R. (2008). Structural equation modelling: Guidelines for determining model fit. Electron. J. Bus. Res. Methods 6, 53–60.

[ref9002] Hu L. Bentler P. M. (1999). Cutoff criteria for fit indexes in covariance structure analysis. Structural Equation Modeling 6, 1–55.

[ref16] Jabeen R. Khan R. Ellahi A. Begum S. (2021). Investigating the impact of performance appraisal justice on job performance and OCB of employees: path through work engagement. Humanit. Soc. Sci. Rev. 9, 315–325. doi: 10.18510/hssr.2021.9332

[ref17] Jeon D. Jeong J. (2024). Does performance appraisal fairness alleviate police officers’ organizational silence? Considering the moderating effect of trust. Int. J. Law Crime Justice 79:100703. doi: 10.1016/j.ijlcj.2024.100703

[ref18] Kahrıman M. Avcı S. Aladağ Z. (2021). Investigation of the perception of organizational justice and the effect of mobbing on job satisfaction and intention to leave of employment. Eur. J. Sci. Technol. 28, 162–171. doi: 10.31590/ejosat.994387

[ref19] Kang I. Kim N. Loh W. Bichelmeyer B. (2021). A machine-learning classification tree model of perceived organizational performance in U.S. federal government health agencies. Sustainability 13:10329. doi: 10.3390/su131810329

[ref20] Kebede S. Wang A. (2022). Organizational justice and employee readiness for change: the mediating role of perceived organizational support. Front. Psychol. 13:806109. doi: 10.3389/fpsyg.2022.806109, 35369209 PMC8965650

[ref9001] Kline R. B. (2016). Principles and Practice of Structural Equation Modeling (4th ed.). New York, NY: Guilford Press.

[ref21] Lapuz R. b. Macaculop A. B. Rosario M. L. S. D. Diaz J. A. M. Tuapen S. M. C. Pascual M. P. (2021). Effectiveness of the public financial management assessment tool or (PFMAT) in strengthening the financial capability of local government unit in Talavera, Nueva Ecija. Int. J. Adv. Eng. Manag. Sci. 7, 52–61. doi: 10.22161/ijaems.76.6

[ref23] Marzec I. (2023). The role of LMX and organizational climate in improving performance and effectiveness of local public administration: evidence from the Metropolis GZM. Region. Develop. Region. Policy. 14, 149–160. doi: 10.14746/rrpr.2023.63.09

[ref24] Mok M. Leong Y. (2021). Factors affecting the effectiveness of employees’ performance appraisal in private hospitals in Malaysia. Int. J. Bus. Soc. 22, 257–275. doi: 10.33736/ijbs.3174.2021

[ref25] Moon K. K. Lim J. K. Kim J. S. (2024). Examining the effect of organizational justice on turnover intention and the moderating role of generational differences: evidence from Korean public employees. Sustainability 16:2454. doi: 10.3390/su16062454

[ref26] Mostafa A. M. S. Gould-Williams J. Bottomley P. (2015). High-performance human resource practices and employee outcomes: the mediating role of public service motivation. Public Adm. Rev. 75, 747–757. doi: 10.1111/puar.12354

[ref27] Osei-Kojo A. (2016). E-government and public service quality in Ghana. J. Public Aff. 17:e1620. doi: 10.1002/pa.1620

[ref28] Ouabi Z. Douayri K. Barboucha F. Boubker O. (2024). Human resource practices and job performance: insights from public administration. Societies 14:247. doi: 10.3390/soc14120247

[ref29] Ruijer E. Porumbescu G. Porter R. Piotrowski S. (2022). Social equity in the data era: a systematic literature review of data-driven public service research. Public Adm. Rev. 83, 316–332. doi: 10.1111/puar.13585, 40046247

[ref30] Saoula O. Fareed M. Hamid R. A. Esmail Abu Al-Rejal H. M. Ismail S. A. (2019a). The moderating role of job embeddedness on the effect of organisational justice and organisational learning culture on turnover intention: a conceptual review. Humanit. Soc. Sci. Rev. 7, 563–571. doi: 10.18510/hssr.2019.7267

[ref31] Saoula O. Fareed M. Ismail S. A. Husin N. S. Hamid R. A. (2019b). A conceptualization of the effect of organisational justice on turnover intention: the mediating role of organisational citizenship behaviour. Int. J. Finan. Res. 10, 327–337. doi: 10.5430/ijfr.v10n5p327

[ref32] Shonhadji N. (2022). Antecedents of whistleblowing intentions: evidence in local government agencies. AKRUAL 14, 63–79. doi: 10.26740/jaj.v14n1.p63-79

[ref33] Sökmen A. (2020). Organizational justice, organizational trust, job satisfaction, and intention to leave relationship: a research on public servants. Third Sector Soc. Econ. Rev. 55, 2651–2663. doi: 10.15659/3.sektor-sosyal-ekonomi.20.11.1492

[ref34] Stankevičiūtė Ž. Savanevičienė A. (2021). Linkage between leaders’ behaviour in performance management, organizational justice and work engagement in public sector. Economies 9:15. doi: 10.3390/economies9010015

[ref35] Tantardini M. (2022). Organizational social capital and performance information use: the mediating role of public service motivation. State Local Gov. Rev. 54, 202–220. doi: 10.1177/0160323X221113366

[ref36] Tsyhaniuk D. Akenten C. W. (2025). The nexus between employee budget participation and employee performance. Econ. Sustain. Bus. Pract. 2, 61–69. doi: 10.21272/esbp.2025.1-08

[ref37] Whitebread G. Dolamore S. Stern B. (2022). Quantitative intersectionality: imperatives and opportunities for advancing social equity. Public Adm. Rev. 83, 117–129. doi: 10.1111/puar.13555, 40046247

[ref38] Zelnick J. Goodkind S. Kim M. E. (2021). “It would be foolish to pretend that our jobs aren't political”: social workers organizing for power in the nonprofit sector. Affilia 37, 5–12. doi: 10.1177/08861099211065926, 42010384

[ref39] Zhou L. Tetgoum Kachie A. D. Xu X. Quansah P. E. Epalle T. M. Ampon-Wireko S. . (2022). COVID-19: the effects of perceived organizational justice, job engagement, and perceived job alternatives on turnover intention among frontline nurses. Front. Psychol. 13:920274. doi: 10.3389/fpsyg.2022.920274, 36148111 PMC9486381

