# Supplementary material for: The mediating role of organizational justice in the relationship between cost consciousness and resource efficiency among human service professionals
Source: Front Psychol. 2026 Oct 2;17:1903704. doi: 10.3389/fpsyg.2026.1903704 (PMC13671923; doi:10.3389/fpsyg.2026.1903704)
Supplement: Supplementary file 1 [file Data_Sheet_1.pdf]

# Appendix A: Measurement Instruments

*This appendix presents the complete item wording for the three original scales developed for this study. All items were rated on a 5-point Likert scale ranging from 1 (Strongly Disagree) to 5 (Strongly Agree). Items were originally administered in Turkish; the English wording below is provided for reporting purposes.*

## A.1 Cost Consciousness Scale (CC)

| Item | 1 | 2 | 3 | 4 | 5 |
|---|---|---|---|---|---|
| CC1. I have a general awareness of the cost structure of the services provided by my institution. | | | | | |
| CC2. I can anticipate that a service may generate unnecessarily high costs. | | | | | |
| CC3. I behave consciously to prevent the wastage of institutional resources. | | | | | |
| CC4. I take the cost–benefit balance into account when making decisions. | | | | | |
| CC5. I believe that cost management can improve service quality. | | | | | |
| CC6. I think that the sustainability of the institution will be strengthened through the efficient use of resources. | | | | | |
| CC7. I observe that cost planning is part of strategic decisions in my institution. | | | | | |
| CC8. I believe that accessibility to cost data instills a sense of responsibility in employees. | | | | | |
| CC9. I believe that staff should be regularly informed about the cost of resources. | | | | | |
| CC10. I can identify methods that could achieve cost savings within my area of duty. | | | | | |

## A.2 Resource Efficiency Scale (RE)

| Item | 1 | 2 | 3 | 4 | 5 |
|---|---|---|---|---|---|
| RE1. I attach importance to carrying out work processes efficiently in terms of time, effort, and resources. | | | | | |
| RE2. I consider achieving the same outcome with fewer resources to be an institutional responsibility. | | | | | |
| RE3. I take care to complete my tasks in the shortest possible time and in an effective manner. | | | | | |
| RE4. I believe that unnecessary use of institutional resources negatively affects service quality. | | | | | |
| RE5. I am willing to offer improvement suggestions aimed at increasing efficiency. | | | | | |
| RE6. I observe that planned and systematic progress in resource use increases efficiency. | | | | | |

| | | | | | |
|---|---|---|---|---|---|
| RE7. I believe that repetitive or non-functional steps in work processes should be reduced. | | | | | |
| RE8. I find it necessary to increase practices that support the effective use of resources in the institution. | | | | | |
| RE9. I believe that the efficient use of time is a determining factor in institutional success. | | | | | |
| RE10. I believe that an efficiency-oriented work culture will provide long-term benefit to the institution. | | | | | |

## A.3 Organizational Justice Perception Scale (OJP)

| Item | 1 | 2 | 3 | 4 | 5 |
|---|---|---|---|---|---|
| OJP1. I think that the distribution of opportunities and resources among employees in my institution is balanced. | | | | | |
| OJP2. I observe that duties and responsibilities are assigned according to fair criteria. | | | | | |
| OJP3. I believe that performance-based evaluations are conducted objectively. | | | | | |
| OJP4. I think that employees' opinions are taken into account in decision-making processes. | | | | | |
| OJP5. I observe that institutional policies are applied equally to all personnel. | | | | | |
| OJP6. I experience that managers' attitudes toward employees are respectful and inclusive. | | | | | |
| OJP7. I think that communication processes in the institution are carried out openly and transparently. | | | | | |
| OJP8. I observe that feedback provided to employees is constructive and developmental in nature. | | | | | |
| OJP9. I believe that the institution embraces a work culture based on the principle of justice. | | | | | |

A
